# On the Inefficiency of the Act of Vomiting in Removing Arsenic from the Stomach

**Published:** 1827-03

**Authors:** James Scott

**Affiliations:** Surgeon.


### VOMITING.

*On the Inefficiency of the act of Vomiting in removing Arsenic from the Stomach.* By James Scott, Surgeon.

I was called, a short time ago, to Susan Walmer, a young woman, ætatis sixteen, residing at No. 5, William-street, Newington; the messenger informing me that she was labouring under violent pain of the stomach, suspected to arise from poison. I found her writhing under great torture, which she referred to the epigastric region; her tongue loaded with a thick buffy coat; her breathing quick and oppressed; her pulse imperceptible; her body cool, the extremities icy cold. She complained of a "burning heat" in the mouth, and a sensation of constriction of the fauces, (or "choking," as she termed it,) amounting almost to suffocation. Her thirst was insatiable, and she called incessantly for water. She had vomited severely for two hours, and her bowels had been frequently purged with dark and offensive motions. I asked her what she had taken? to which she replied, fifteen pennyworth of laudanum and half a teacupful of arsenic.

The matters she had vomited had not been preserved, and I had no means, therefore, of knowing what had been thrown up; but, aware of the extreme tenacity with which arsenic adheres to the mucous membrane of the stomach, I considered it my duty to cleanse this organ mechanically, notwithstanding the exhausted state of the patient indicated the probable approach of dissolution. Two quarts of warm water were injected with the stomach-pump, and withdrawn; when two quarts more were thrown in and withdrawn, in a similar manner. I was proceeding to inject a further quantity, when a change of the unhappy patient's countenance and appearance warned me that death was nearer at hand than the symptoms had at first foreboded. I therefore withdrew the tube, caused her to be laid into bed, and she expired ten minutes afterwards, having survived the fatal dose about six hours.

By an officious interference of some persons about me, the portion of fluid first withdrawn from the stomach had been thrown away; but, on inspecting that of the second operation, I discovered a pulverulent precipitate (which I subsequently ascertained to be arsenious acid); very inconsiderable in quantity however, amounting to a few grains only.

On the following morning I opened the abdomen, and, having put a ligature around the cardiac and pyloric extremities of the stomach, I separated the organ, and removed it to my house for examination. The inspection being undertaken rather with a view of furnishing evidence of the cause of the patient's death, than of tracing the morbid effects of the poison, and my leisure not allowing me an investigation further than was necessary to satisfy judicial inquiry, the result has not enabled me, probably, to furnish any new fact involved in the phenomena of poisoning by arsenic; my object in this communication being to engage professional attention to a striking fact, important in its relation to therapeutical agency.

The stomach, on being opened, was found to contain about twenty ounces of fluid, which had been injected previously to the death of the patient. Upon the removal of this fluid, the surface of the stomach presented universally a bright vermilion blush, with patches of a brownish red scattered here and there, but chiefly on the posterior lateral surface, in the pyloric half of the stomach. These patches were somewhat pulpy, rather loose in texture and adhesion, more glossy than the surrounding parts, and gelatinous in appearance. In fine, they were portions of the mucous membrane in a state of disorganisation, and might be detached by being pinched between the finger and thumb, leaving the muscular coat denuded. *Near the small extremity of the stomach, lay two masses of powdered arsenic, enveloped in a sort of reddish jelly*, which doubtless consisted of the mucous membrane disorganised by the contact of the poison. These were scraped off with a spoon, and the powder separated by repeated washing in cold water. I did not weigh it, but I should guess the quantity of arsenic to have been at least half an ounce.

Here is the point to which I would direct the attention of the medical practitioner. Vomiting, assisted by copious dilution, *during two hours*, had not detached the arsenical powder from the surface of the stomach; and so entangled and blended was it with the softened mucous membrane that I believe no action of the organ itself could have separated it. The injection of a strong current of fluid, by means of Read's stomach-syringe, failed also to detach the mineral: so small, indeed, was the portion of arsenic washed up with the liquid, that, as I remarked before, only a few grains subsided in the vessel in which it was received and allowed to rest; and the chemical tests which I employed gave but very faint traces of arsenic in solution: the sparing solubility of arsenic in water may account, probably, for the latter circumstance.

From these facts, I think that the following conclusion may be adduced:—That the efficacy of emetics in dislodging arsenic from the stomach is confined to a limited time after the poison has been swallowed; but what may be its extent, or at what period the disorganisation of the stomach commences, which agglutinates and fixes the mineral to the surface of the organ, I have not had sufficient experience to ascertain; it being probably determined by the quantity swallowed, by the quality and volume of ingesta, and perhaps by other accidental circumstances.

In the present case, the stomach contained no solid matters, the patient having eaten no food through the day.

In the absence of positive information upon this point, would it not be the safest practice to resort at once to the use of the stomach-pump in these cases? As an indirect reply, I must be allowed to express my conviction that, had I been earlier in attendance upon this unfortunate person, (such is the force of the fluid propelled by the syringe I employed, a power which gives it a distinguishing superiority to any other,) I should have used it successfully. And here I will take leave to notice an opinion that has been given of the inexpediency of the pump in emptying the stomach, from a notion that the operation might be performed by a tube only, upon the syphonic principle: the case I have related is a sufficient commentary upon such a tenet.

I have to state in conclusion, that I ascertained the poisonous substance to be arsenic by a concurrence of all the tests which were employed, as lime-water, nitrate of silver, ammoniuret of copper; and, lastly, by its reduction to a metallic state.

30, *Newington Causeway, Borough;*
*August* 1826.

